# Physiotherapist's Management of Suspected Cauda Equina Syndrome in the United Kingdom: A National Survey

**DOI:** 10.1002/pri.70182

**Published:** 2026-03-17

**Authors:** Rob Tyer, Nick Livadas, Robert Hogg

**Affiliations:** ^1^ University of Sunderland Sunderland UK; ^2^ Cora Health Teesside University Middlesbrough UK

**Keywords:** cauda equina Syndrome, CES, clinical decision making, emergency referral, musculoskeletal, physiotherapy

## Abstract

**Background and Purpose:**

Cauda Equina Syndrome (CES) is a rare but serious spinal condition requiring urgent diagnosis and management. Physiotherapists in UK musculoskeletal (MSK) services increasingly encounter suspected CES cases, but little is known about their clinical decision‐making and referral practices. This study aimed to examine the clinical decision‐making processes of UK physiotherapists when presented with three clinical vignettes of suspected CES and to identify themes in referral handover methods when emergency care is deemed necessary.

**Methods:**

An online survey featuring three clinical case vignettes was distributed to UK physiotherapists. Respondents were asked about their referral decisions, clinical reasoning, and preferred methods of handover to Accident & Emergency (A&E) services. Thematic analysis was used to identify common clinical reasoning patterns and referral practices.

**Results:**

The presence of altered bladder function was the strongest predictor of referral to A&E, consistent with previous literature. Sexual dysfunction was also a significant referral factor when present. Themes for not referring included recent A&E attendance, plans for in‐clinic assessment, specialist consultation, and requesting urgent MRI scanning. A range of handover methods were reported, with written letters and direct phone calls being the most common. Variation in clinical decision‐making was evident, particularly regarding timing and urgency of investigations, reflecting some uncertainty around guideline interpretation.

**Discussion:**

Findings demonstrate variability in UK physiotherapists' approaches to suspected CES management, with some respondents opting for community assessment or delayed imaging despite CES's urgent nature. Findings emphasise the need for clearer, unified guidelines on referral pathways and handover methods to reduce delays and improve patient outcomes. Future research should explore the impact of recent guideline updates and include perspectives from other healthcare professionals involved in CES care.

## 1 Introduction

Cauda Equina Syndrome (CES) is a rare but serious condition manifesting from compression of the Cauda Equina (CE) nerve roots in the lumbosacral spine (Ahad et al. 2015). Left untreated, it can result in permanent loss of function of the bladder, bowel, sexual organs, saddle sensation and lower limb power (GIRFT. Spinal Surgery 2023). Diagnosis requires subjective and/or objective symptoms combined with compression of the CE nerve roots evident with Magnetic Resonance Imaging (MRI) (Todd 2015). The reported prevalence in United Kingdom (UK) secondary and tertiary specialist spinal units is reported to be 15.6% (Fountain et al. 2019). It represents 2% of those with confirmed lumbar disc herniations (Greenhalgh et al. 2016) but is present in < 1% of all low back pain (LBP) cases (Greenhalgh et al. 2018). Despite variance in international guidelines, acute CES is widely recognised as a medical emergency requiring surgical decompression within 48 h of symptom presentation (Germon et al. 2015; Srikandarajah et al. 2015; Todd and Dickson 2016). However, in the absence of readily accessible MRI scanning, an ongoing debate persists regarding the diagnostic utility of clinical assessment and other imaging methods.

The predictive value of objective tests such as bulbocavernosus reflex (BCR), post‐void residual (PVR) scanning, and anal/rectal tone assessment for guiding same day MRI referrals in suspected CES remains conflicting. While some studies report high sensitivity, specificity, and negative predictive values (NPV) for BCR (100%) and PVR > 200 mL (NPV 96%–98.7%) in specialised settings (Katzouraki et al. 2020; Zusman et al. 2022), larger datasets and guidelines challenge their reliability (Woodfield et al. 2023; Lopez et al. 2024). Current guidance (GIRFT. Spinal Surgery 2023) and evidence (Gooding et al. 2013; Angus et al. 2022) caution against relying on these tests due to operator variability, low accuracy, and inconsistent predictive power across diverse clinical settings.

The Getting It Right First Time (GIRFT) programme aims to address unwarranted variation in clinical practice within the National Health Service (NHS) (The King's Fund). According to GIRFT, 23% of all litigation cases for spinal surgery in England were related to cases of CES (GIRFT. Spinal Surgery 2023). Therefore, the implications of delayed care represent significant financial burdens to the health service, with an estimated cost of over £186 Million approximately 2008–2018 (NHS Resolution 2020). Published litigation data regarding physiotherapists between 2006/7–2016/17 reported 119 successful claims awarded to people with CES (Beswetherick 2019). The most contemporary data specifically investigating claims involving physiotherapists in the UK across all sectors, identified 51 CES claims between 2012–2021, with payouts ranging from £200,000–£1.5Million (Yeowell et al. 2022). Although the financial implications are important to note, they do not compare to the devastation this condition can have on the quality of life of those with it (Korse et al. 2017).

Physiotherapists are trained to question those with LBP in a way which would increase or decrease their suspicion of a CES presentation using red flag questions (Greenhalgh et al. 2016). Red flag questions for serious spinal pathology have low sensitivity and higher specificity, but overall offer low predictive value as standalone measures (Premkumar et al. 2018). The International Framework for Orthopedic Manual Physical Therapists (IFOMPT) position statement, designed to support clinical reasoning and management of cases presenting with spinal red flags, encouraged the use of context alongside these questions (Finucane et al. 2020). To date, there are no published data to our knowledge on the reasoning process or behaviour of community based musculoskeletal (MSK) physiotherapists when faced with suspected CES. This clinical vignette aims to canvas the decision making of UK based physiotherapists when dealing with 3 fictitious, but clinically common presentations of patients with LBP.

Study aims:

This study aims to:

1. Examine the clinical decision‐making processes of UK physiotherapists' when presented with three different clinical vignettes.
2. Establish themes within handover methods used when emergency care is considered the appropriate option.

## 2 Methods

This section follows the Standards for Reporting Qualitative Research (SRQR) guidelines recommended by The EQUATOR Network (Enhancing the QUALity and Transparency Of health Research—See Supporting Information S1: Appendix 1) (O’Brien et al. 2014).

### 2.1 Design

Mixed methods design was employed, collecting quantitative (demographic and decision frequencies) and qualitative (open ended responses to vignettes) data through an online survey of physiotherapists working in the UK with a caseload of people with LBP.

### 2.2 Participants

Participants were required to be a fully qualified and registered with the Health and Care Professions Council, currently practicing in MSK care within the UK. Student physiotherapists, other allied health professionals, medical doctors, and other providers of MSK care were excluded from this study.

### 2.3 Ethics and Declaration

Ethical approval was granted by the University Ethics Committee (University of Sunderland, 013539) for this project.

### 2.4 Survey Design

Two Advanced Practice Physiotherapists (RT and NL) constructed three case scenarios reflecting common primary care presentations. The researchers acknowledge that their professional experiences and assumptions on clinical decision making may have shaped the study design. To mitigate this bias and thus increase face validity, the vignettes were shared with three Advanced Practice Physiotherapists, regarded as experts in the field of serious spinal pathology. They were asked to confirm whether the cases accurately represent patients with LBP along with suspicious but unclear features of CES. Feedback regarding the clarity of questions and the appropriateness of the information included informed amendments made to the design.

This open survey was designed by all three authors and included a mixture of open and closed questions. These questions aimed to establish decision‐making themes of the participants, and how the presenting symptoms within the vignettes influenced these decisions. A piloting phase was completed to refine the survey design, which accounted for 8 sets of responses, which were excluded from the data.

### 2.5 Recruitment

Recruitment occurred between November 2022 and February 2023, ahead of the updated GIRFT guidelines release (2023). A snowballing approach was used to identify appropriate online forums where MSK physiotherapists engage in clinical discussion (iCSP, Facebook Special Interest Groups, X [formally twitter], LinkedIn). In addition, several MSK special interest groups acted as gatekeepers to disseminate the survey links to their members.

### 2.6 Consent

Participation in the survey was voluntary and anonymous. A participant information leaflet was available for all participants, outlining the process of the study (see Supporting Information S1: Appendix 2). Withdrawal from the study was possible up until the submission of the survey data, after which point, withdrawal was not possible due to participant anonymity. No financial incentives were offered for participation.

### 2.7 Data Collection

Following the case presentation, participants were asked a series of open‐ended questions aimed at understanding the clinical reasoning behind their actions for each case (see Supporting Information S1: Appendix 3 for the questionnaire). The results were exported from Qualtrics (Provo, UT, Available at https://www.qualtrics.com/) and analysed using Microsoft Excel (Microsoft Corporation, 2018. *Microsoft Excel*, Available at https://office.microsoft.com/excel).

### 2.8 Data Analysis

The survey included the same eight questions for each vignette to allow for inductive content analysis regarding decision making processes (Elo and Kyngäs 2008). Results were exported from Qualtrics to a Microsoft Excel spreadsheet where the qualitative analysis was completed. This focused primarily on the four most frequently cited reasons for not referring each vignette to Accident and Emergency (A&E) Incomplete surveys were excluded to improve response quality and accuracy of the data analysis when comparing outcomes between vignettes. This decision was made because of the use of Cronbach's alpha as a key part of the analysis, which can be susceptible to variations in sample size, from unstable estimates in small samples (Cortina 1993) to overly sensitive significance in large samples (Schmitt 1996). Maintaining a consistent sample size between vignettes seemed prudent, especially as this would then be comparing responses of those that expressed opinions on all three vignettes. Themes were generated from the survey data by RT and NL independently to mitigate potential bias, following which a consensus meeting was held, and final themes were agreed upon.

## 3 Results

**FIGURE 1:**
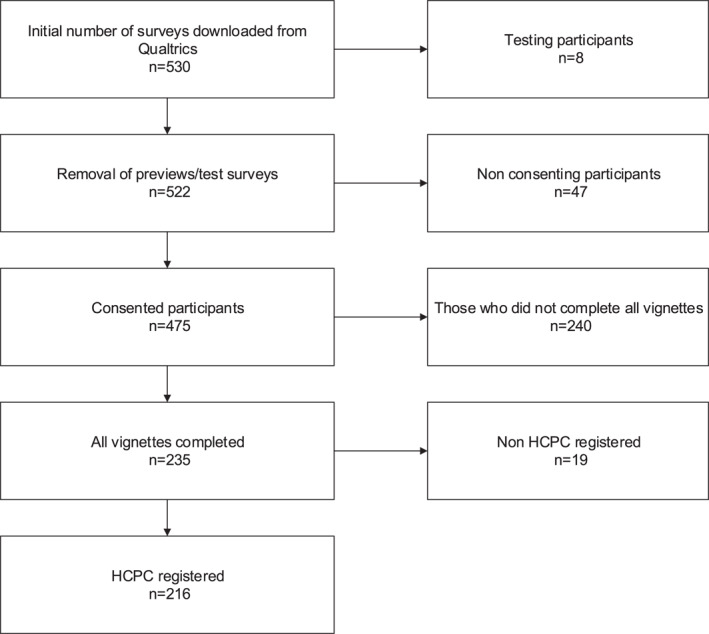
Data cleaning process flow chart.

A total of 475 consented to participate, 235 of whom completed all vignettes, 216 of whom were confirmed as Health and Care Professions Council registered physiotherapists (see Figure 1 for data cleaning process flow chart).

### 3.1 Demographic Data

Most respondents were from Northwest England accounting for 15.7% of completed surveys (Table 1). Physiotherapists with 11–15 years of experience were most highly represented (Table 1). Two thirds of the responses were from those working in an NHS service (Table 1), and 60.2% of responses considered themselves to have a special interest in spinal conditions (Table 1).

**TABLE 1:** Participant characteristics.

Characteristic	Category	*n*	%
Geographic region	Northwest England	34	15.70
	Northeast England	23	10.70
	Scotland	23	10.70
	Southwest England	22	10.20
	London	22	10.20
	Southeast England	21	9.72
	Yorkshire & The Humber	16	7.41
	West Midlands	16	7.41
	East of England	14	6.48
	Northern Ireland	9	4.17
	Wales	9	4.17
	East Midlands	7	3.24
Years qualified	5 years and under	20	9.26
	5–10 years	31	14.40
	11–15 years	48	22.20
	16–20 years	35	16.20
	21 years and over	82	38.00
Practice sector	NHS	143	66.20
	Independent provider of NHS services	37	17.10
	Private practice	27	12.50
	Occupational health	6	2.78
	Sports physiotherapy	2	0.93
	Missing	1	0.46
Spinal special interest	No	130	60.20
	Yes	86	39.80

### 3.2 Vignette Clinical Content

Table 2 presents the clinical information available to the respondents at the time of completing the survey.

**TABLE 2:** Vignette information.

Consultation	Vignette 1	Vignette 2	Vignette 3
	Telephone consultation	Face to face consultation	Face to face consultation
Background	Miss B is a 19‐year‐old carer, who has developed back pain and bilateral leg pain 2 days ago. It started immediately after catching an elderly resident who fell out of bed during a bed slide. She has never had back pain before and feels very disabled by the levels of pain and lack of mobility that she has. Miss B has not long returned to work after 8 months maternity leave, and she feels unable to pick up her daughter, so she is relying on family for help. The current pain is described to you as; Constant with varying levels of pain depending on movement. Pain and tingling in both legs at the same time. Difficulty sleeping due to pain and young child in the house. Finding climbing the stairs difficult due to pain and effort level needed.	Mr S is a 28‐year‐old mechanic, who attends with a 9‐week history of low back pain following lifting a car battery from an engine and placing it on the floor. He was struck by severe pain and couldn’t move; his workmates then called for an ambulance. Mr S was seen in A&E and was given Oramorph but discharged without assessment or investigation. Mr S has been unable to work since and cannot find a position of ease. He has spent his time in bed, in his chair or with pacing the floor due to pain in all positions after 15 min. The pain is severe, radiating down the right leg to his big toe for the last 9 weeks. He is struggling to sleep despite medication and wakes up whenever he rolls over.	Mrs J is a 65‐year‐old, who attends with a history of low back pain which comes and goes over the last 5 years. Previous episodes have had associated left leg pain from buttock to heel. This episode of low back pain started 4 weeks ago, with vigorous coughing after a chest infection. There was a sudden onset of severe pain, with radiation of left leg pain from buttock to heel, and over the last 4 weeks this has been progressively worsening in severity and now include the right leg pain from buttock to heel for the last 5 days. She reports bilateral foot “numby feelings” and like “I am walking on sponges” subjectively. The pain is constant, but the severity changes with prolonged static positions (sitting, standing, lying), until she moves. There is severe “electric shock” pain with coughing, and Mrs J cannot stand fully upright due to leg pain. Rolling over in bed is provocative of her pain, but there is no particular morning pattern.
	She attended A&E the next day, where a digital rectal exam and bladder scan was completed (as had dribble incontinence, with increased frequency and not feeling like she has fully voided). Told things were normal and discharged to care of GP and recommended the GP consider an MRI scan due to a “nerve problem”.		
Past Medical History	Symphysis pubis dysfunction during recent pregnancy 4th degree perineum tearing during labour Obesity Osteochondroma left distal femur Constipation	Post‐traumatic Stress Disorder – ex military 2012 Asthma Psoriasis irritable bowel syndrome Sportsman’s hernia repair	COPD Type 2 diabetes Hypothyroidism Bladder sling surgery 4 years ago Total hysterectomy 15 years ago due to ovarian cysts Anxiety
Drug History	Naproxen 220mg every 8–12 hours Tramadol 100mg every 4–6 hours Movicol 1 sachet daily.	Amitriptyline 10mg × 1 per day Sertraline 100mg daily	Salbutamol Metformin Gliclazide Oxybutynin Citalopram Recently started on Tramadol (50 mg, 4 hourly) and Amitriptyline (10 mg)
Social History	Smoker Teetotaller Lives at home with mother and 1 × child. No sports/hobbies.	Lives at home with his wife and 2 children, 8 months old and 2 years old Nonsmoker Drinks 8 cans of lager on the weekend Full time mechanic Football 2 x week	Lives alone since her husband died 5 years ago Smoker—20 per day for the last 35 years Teetotaller Work—Recently retired School Cook. Hobbies/sports—Walks her dog daily, 20 minutes per day Other—Helps to look after 3 grandchildren at home, 2, 6, and 13
Bladder	Sudden urge to urinate. Can’t make it to the toilet on time over the last 2 days and has dribble incontinence before she can sit down. Increased frequency of urination from 6 times per day to 12+, feeling like she cannot fully empty her bladder.	For the last 5 weeks. When urinating, has to go back a second time to finish emptying his bladder. Prior to accident never had trouble.	Mrs J also reports that since her low back pain and leg pain have become worse, she cannot make it to the toilet on time and occasionally has started urinating before she gets there. She can wake in the morning and note some wetness in her underwear.
Bowel	Long term use of Movicol since birth of child. No changes since onset of low back pain.	Irritable Bowel Syndrome has actually been less of a problem, not going as often (previously 5 times per day, to now 1–2 per day).	She has normal bowel function.
Saddle	Difficult to answer as there has been altered sensation since perineum tearing. No significant noticeable changes.	Numb around right testicle and inner thigh since “sportsman’s hernia repair” 3 months ago. No changes in that time.	There is an odd feeling when she wipes after urinating and she thinks she can feel a prolapse, similar to when she had her sling surgery.
Sexual	Not sexually active at present.	Erectile difficulties since discharge from the military with Post Traumatic Stress Disorder, however, struggling to reach climax over the last 8 weeks, possibly due to pain during intercourse.	Not sexually active at present.
Other	Bilateral leg pain and paraesthesia.	Night pain as described above.	There is no slapping of the foot, but she feels like she is walking on sponges, but denies upper limb symptoms. Night pain. Progressive unilateral to bilateral leg symptoms.

### 3.3 Level of Suspicion for CES

Of the 216 respondents, a significant proportion felt that each case study warranted referral to A&E for suspected CES. For vignette 1, this was 57.9% (*n* = 125) of respondents, for vignette 2, 31.5% (*n* = 68) of respondents and for vignette 3, 62.5% (*n* = 135) of respondents.

For each vignette in the questionnaire, a multiple regression analysis was performed using IBM SPSS statistics software (IBM 2025), with the dependent (or target) variable being the suspicion of CES and the independent variables being the 8 listed symptoms. Table 3 lists the regression coefficients, ordered in size, with positive coefficients indicating an increase in suspicion of Cauda Equina for a one‐unit increase of the symptoms on the questionnaire scale. In all three vignettes, ‘Altered Bladder Function’ emerged as the strongest predictor of Suspicion of CES.

**TABLE 3:** Level of CES suspicion.

Vignette 1		Vignette 2		Vignette 3	
Symptom	Regression coefficient	Symptom	Regression coefficient	Symptom	Regression coefficient
Altered bladder function	0.70^a^	Altered bladder function	0.60^a^	Altered bladder function	0.68^a^
Bilateral leg symptoms	0.37^a^	Sexual dysfunction	0.30^a^	Saddle sensation changes	0.30^a^
Progressive symptoms	0.07	Progressive symptoms	0.11	Progressive symptoms	0.27^a^
Level of pain	0.06	Saddle sensation changes	0.06	Level of pain	0.21^a^
Saddle sensation changes	0.01	Altered bowel function	0.06	Bilateral leg symptoms	0.10
Sexual dysfunction	0.00	Impact on sleep	0.02	Altered bowel function	0.02
Impact on sleep	−0.01	Bilateral leg symptoms	0.00	Impact on sleep	−0.07
Altered bowel function	−0.04	Level of pain	−0.03	Sexual dysfunction	−0.15

^a^ Statistically significant at 0.05 level.

### 3.4 Physical Assessment

**FIGURE 2:**
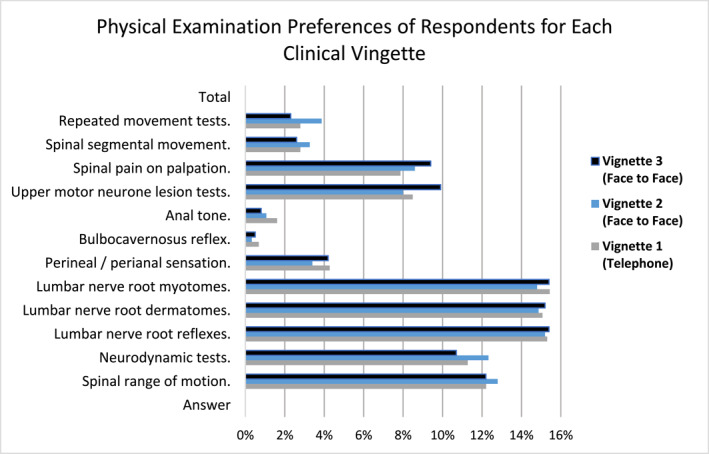
Physical examination options selected per vignette.

Question four of each vignette asked, ‘If you were going to physically examine this patient, what would your assessment consist of?’. Those who felt it important to assess each of the vignettes could select more than one assessment option. Figure 2 presents the percentage prevalence of the assessment options selected by this group for each vignette.

When ranked in order of most to least commonly selected option, all three vignettes shared the same first five most selected assessment techniques in the same order (lumbar myotomes, lumbar nerve root reflexes, lumbar dermatomes, spinal range of motion, and neurodynamic assessment). The sixth and seventh most common were upper motor neurone assessment followed by spinal pain on palpation in vignettes 1 and 3 but in opposite order for vignette 2. Perianal sensation, spinal segmental movement and repeated spinal movements were the next most common in that order for vignettes 1 and 3, with vignette 2 listing repeated movements above perianal sensation and spinal segmental movement. The assessment techniques with the lowest frequency of selection were abdominal/visceral palpation, anal tone and bulbocavernosus reflex. All assessment options were selected as something that a participant may assess in each vignette case.

### 3.5 Management Plan

Question 5 of the survey for each vignette asks, ‘What would your management plan be today?’. This question allowed multiple options to be selected to account for situations which required more than one action, the results of which can be seen in ranked order in Table 4.

**TABLE 4:** Management plan for each case vignette, ranked.

Rank	Vignette 1	Count (%)	Vignette 2	Count (%)	Vignette 3	Count (%)
1	Ask the patient to attend A&E today	186 (54.39%)	Ask the patient to attend A&E today	86 (31.97%)	Ask the patient to attend A&E today	138 (62.12%)
2	See the patient yourself	48 (14.04%)	Request an urgent MRI	68 (25.28%)	See the patient yourself	20 (9.01%)
3	Request an emergency MRI	44 (12.87%)	See the patient yourself	62 (23.05%)	Request an urgent MRI	17 (7.66%)
4	Request an urgent MRI	41 (11.99%)	Direct referral to spinal services	25 (9.29%)	Direct referral to spinal services	15 (6.76%)
5	Direct referral to spinal services	18 (5.26%)	Request a routine MRI	13 (4.83%)	Request an emergency MRI	10 (4.50%)
6	Ask the patient to attend their nearest walk‐in centre	2 (0.58%)	Ask the patient to attend their GP	6 (2.23%)	Ask the patient to attend their GP	9 (4.05%)
7	Ask the patient to attend their GP	2 (0.58%)	Request an emergency MRI	6 (2.23%)	Refer for an X ray	6 (2.70%)
8	Request a routine MRI	1 (0.29%)	Ask the patient to attend their nearest walk‐in centre	2 (0.74%)	Ask the patient to attend their nearest walk‐in centre	4 (1.80%)
9	Refer for an Xray	0 (0.00%)	Refer for an Xray	1 (0.37%)	Request a routine MRI	3 (1.35%)
Total		342 (100%)		269 (100%)		222 (100%)

The most frequently selected option was to refer each patient to the local A&E department, followed by either opting to see the patient themselves, requesting an urgent MRI, requesting an emergency MRI, or directly referring to spinal services.

### 3.6 Handover Methods

Table 5 outlines the preferred handover method of the respondents, with the most frequently cited methods being the provision of a written letter (either physical or electronic), followed by a telephone call to A&E accompanied by a supporting letter. The least common responses were to ask the patient to communicate the reasons for referral directly with A&E or for the physiotherapist to send clinical records directly to Spinal Services. These relatively small numbers may reflect the nature of the survey, which allowed respondents to select multiple options. As there was a list of options provided, some may have selected all as a ‘just in case’ approach to answering.

**TABLE 5:** Handover methods when referring to A&E.

Rank	Vignette 1 Handover method	Count (%)	Vignette 2 Handover method	Count (%)	Vignette 3 Handover method	Count (%)
1	Provide written letter as handover (or electronic)	97 (36%)	Provide written letter as handover (or electronic)	42 (35%)	Provide written letter as handover (or electronic)	56 (34%)
2	Telephone handover plus written letter	64 (24%)	Telephone handover plus written letter	26 (22%)	Telephone handover plus written letter	45 (27%)
3	Provide completed service developed written pro forma	35 (13%)	Other	16 (13%)	Provide completed service developed written pro forma	17 (10%)
4	Other	25 (9%)	Provide completed service developed written pro forma	15 (13%)	Other	9 (15%)
5	Provide MACP (or alternative) CES card to demonstrate your reason for referral	24 (9%)	Telephone handover to triage clinician	8 (7%)	Provide MACP (or alternative) CES card to demonstrate your reason for referral	14 (8%)
6	Telephone handover to triage clinician	17 (6%)	Provide MACP (or alternative) CES card to demonstrate your reason for referral	7 (6%)	Telephone handover to triage clinician	11 (7%)
7	Complete shared electronic record with spinal services	5 (2%)	Send patient and ask them to verbally communicate reason for attendance	3 (3%)	Complete shared electronic record with spinal services	4 (2%)
8	Send patient and ask them to verbally communicate reason for attendance	4 (1%)	Complete shared electronic record with spinal services	2 (2%)	Send patient and ask them to verbally communicate reason for attendance	3 (2%)
Total		271 (100%)		119 (100%)		165 (100%)

### 3.7 Reasons for Not Sending to A&E: Thematic Analysis

In situations where respondents felt A&E was not appropriate, the top four themes were identified by RT and NL. Combined comparisons were not possible because each vignette contained different themes.

#### 3.7.1 Themes: Vignette 1

The four main themes found for this case study can be seen in Table 6. A third of the total respondents felt that sending Miss B to A&E would not be appropriate as she had already attended and had been discharged without investigation for CES.

**TABLE 6:** ‘If not referring to A&E, please tell us more about your clinical reasoning.’—Agreed themes from vignette 1.

Theme	Example comments	Count (%)
Already been to A&E	V1R17, a physiotherapist working in sport, in Southwest England, with between 16–20 years of experience comments—‘Has already been to A&E. Peri‐anal sensation and tone checked. In absence of any new worsening of symptoms, I think it is reasonable to see how it settles with thorough safety netting as it is still very acute’	24 (32%)
	V1R18, a physiotherapist working in the NHS, in Northwest England with between 5–10 years experience comments—‘Has attended with appropriate tests carried out. Will safety net and give CES card. Then look at pain relief’	
Assess in Physiotherapy	V1R44 an NHS physiotherapist, in Northwest England, with > 21 years experience comments—‘I would review the patient myself and arrange emergency MRI myself, not burden another service’	17 (23%)
	V1R38 an NHS physiotherapist, in Northern Ireland, with > 21 years experience comments—‘Normal rectal exam and bladder scan. If normal myotomes, dermatomes and reflexes I would treat, monitor and review regularly’	
Consult with specialist doctor	V1R20 an NHS physiotherapist, in Southwest England, with > 21 years experience comments—‘Seen in accident and emergency and discharged so sending back to accident and emergency is not a viable option to me. Needs emergency (same day) MRI due to symptoms so I would access this by contacting on call spinal surgeon to discuss’	11 (15%)
	V1R47 an NHS physiotherapist in Scotland with > 21‐year experience comments—‘Our pathway for suspected CES is to call on call neurosurgical registrar’	
	V1R36 a physiotherapist, in East England, with 5–10 years experience, providing NHS services via independent service provider in the East of England comment—‘Direct line to MRI department. Already been accident and emergency and not scanned’	
Urgent/priority MRI	V1R21 an NHS physiotherapist, in Southeast England, with > 21 years of experience felt an MRI was required in this case and comments—‘They have already attended and been discharged, if no change in symptoms and more than 24 hours of symptoms unlikely to change the outcome. MRI is only way to know for sure; I can get MRI scans within days. Safety net patient if any changes in CES to attend Accident and Emergency’	10 (14%)
	V1R26 and NHS physiotherapist, in Northwest England, with 11‐15 years experience felt that community MRI was appropriate, so comments—‘Already seen in accident and emergency and discharged, they haven’t performed an emergency MRI but does dissolve primary care of responsibility so would organise urgent MRI in primary care’	
Remaining themes combined		12 (16%)
Total		74 (100%)

Almost a quarter of the respondents felt that an in‐person Physiotherapy assessment would be appropriate. The urgency of this assessment was not clear, and what information could be gleaned which would alter subsequent decision making.

An almost equal number of clinicians felt that Miss B's case required discussion with a specialist doctor or an urgent/priority MRI (15% and 14% respectively). This would suggest that these clinicians were concerned that the clinical picture could be more than routine back pain/radiculopathy but not concerned enough to refer back to A&E immediately. Some may feel that recent A&E attendance and discharge would prevent them from re‐referring.

#### 3.7.2 Themes: Vignette 2

For this scenario, there were small differences in the numbers of citations for the top emerging themes (Table 7), despite these themes having notable differences in implications for the clinician and patient.

**TABLE 7:** ‘If not referring to A&E, please tell us more about your clinical reasoning.’—Agreed themes from vignette 2.

Theme	Example comments	Count (%)
Outside of timescale	V2R7 a physiotherapist providing NHS services via an independent provider, in Northeast England, with 5–10 years experience comments—‘No change/deterioration within the last 2 weeks so beyond the point of accident and emergency referral as symptoms no longer acute’	56 (28%)
	V2R10 an NHS physiotherapist in Scotland, with > 21 years experience comments—‘Symptoms for more than 4 weeks so not an emergency. After seeing face to face, if concerns would refer as urgent to spinal orthopaedics’	
Don't think it is CES	V2R58 a physiotherapist in Southeast England, working in private Practice, with > 21 years experience comments—‘No reported deterioration in symptoms. Complex presenting subjective history. Would certainly be carefully monitoring plus safety net with CES info. If no improvement with presenting symptoms would refer for MRI if any significant changes this would become urgent’	41 (21%)
	V2R88 a physiotherapist in East Midlands, with 5–10 years experience comments—‘Symptoms appear stable, no evidence or deterioration or progressively worsening in last 4 weeks’	
Urgent/priority MRI	V2R18 a physiotherapist providing NHS services via an independent provider, in Northeast England, with 5–10 years experience comments—‘Symptoms are what I would describe as grey—could have alternative explanations besides CES. Symptoms sound stable so urgent MRI seems appropriate with heavy safety netting’	40 (21%)
	V2R4 an NHS physiotherapist working in Southwest England, < 5 years experience comments—‘Changes to urinary output might be secondary to SSRIs (selective serotonin reuptake inhibitors). Would safety net regarding worsening changes and instead consider urgent MRI’	
Assess in physiotherapy	V2R40 a physiotherapist providing NHS services via an independent provider in West Midlands, with 5–10 years experience comments—‘No progression of symptoms in last 8 weeks described (though would ensure this with patient) so A&E would not assess. Would clarify amitriptyline timeliness as can cause neurogenic bladder symptoms’	23 (12%)
	V2R59 an NHS physiotherapist in Southwest England, with > 21 years experience comments—‘Presents with severe back pain and sciatica with low level change to bladder and bowel function over weeks which appears stable. I have a low level of concern but would wish to carefully safety net and discuss analgesia and perform a baseline neurological examination. The patient is within normal healing times but has not optimised analgesia. His choices are to look at analgesia and allow some time with careful monitoring or to arrange urgent MRI—the priority for this would also be shaped in my mind by his neurological function at assessment’	
Remaining themes combined		32 (18%)
Total		194 (100%)

The most frequently declared reason for not referring Mr S to A&E was that clinicians felt this was no longer within an appropriate timescale for this service (28%). A fifth of respondents (21%, *n* = 41) did not feel Mr S had presented with features in keeping with CES, with a similar number (21%, *n* = 40) feeling that he warranted urgent/priority MRI scanning, highlighting a difference in thresholds held by clinicians.

The final theme had a larger difference than the top three, with 12% of clinicians opting to assess Mr S in clinic rather than referring to A&E.

#### 3.7.3 Themes: Vignette 3

The top four emergent themes for Mrs J's case had a more uniform spread of numbers across each theme (Table 8). A quarter of those not referring Mrs J to A&E based this on the lack of suspicion of CES. However, a fifth of respondents did feel that she would be appropriate for an urgent/priority MRI scan. An almost equal number of clinicians felt that Mrs J's case required assessment within the Physiotherapy clinic or a discussion with a specialist doctor (15% and 14% respectively).

**TABLE 8:** ‘If not referring to A&E, please tell us more about your clinical reasoning.’—Agreed themes from vignette 3.

Theme	Example comments	Count (%)
Don't think it is CES	V3R13 a physiotherapist providing NHS services via an independent provider in Northeast England comments—‘Symptoms likely to be due to previous bladder surgery, should there be some neuro changes on exam may change reasoning’	20 (26%)
	V3R44 an NHS physiotherapist in Northern Ireland, with > 21 years of experience comments—‘age less likely to be cauda equina, bladder sensation more likely prolapse. Warrants MRI due to progressive bilateral leg symptoms and worsening neurology’	
Urgent/priority MRI	V3R4 an NHS physiotherapist in Northeast England, with 10–15 years experience comments—‘Could be discitis or lung cancer with metastases. Patient needs an urgent MRI and bloods’	14 (19%)
	V3R21 an NHS physiotherapist in Northwest England, with > 21 years experience comments—‘I would be suspicious of spinal fracture and possible metastatic cord compression due to onset (coughing) history of smoking and steroid use. No clear cauda equina symptoms but would also refer for an urgent MRI Scan due to the bilateral leg pain, saddle numbness when wiping to exclude CES and metastases’	
Assess in Physiotherapy	V3R28 an NHS physiotherapist in Yorkshire and The Humber, with 16–20 years experience comments—‘Musculoskeletal assessment face to face. Hard neurology’	11 (15%)
	V3R32 a physiotherapist providing NHS services via an independent provider in London, with 5–10 years experience comments—‘Priority face to face appointment for hard neurology to guide pathway. Likely for urgent CATS (intermediate care service) with close CES safety netting’	
Consult with specialist doctor	V3R1 an NHS physiotherapist in Southwest England, with > 21 years experience comments—‘I would bleep the on call spinal orthopaedic consultant to discuss need for surgical assessment unit’	10 (14%)
	V3R42 a physiotherapist providing NHS services via an independent provider in Southeast England, with 11–15 years experience comments—‘As she is having ongoing back and pain no progressive neurological deterioration, I will send her to a specialist spinal surgeon’	
Remaining themes combined		20 (26%)
Total		74 (100%)

## 4 Discussion

To date, existing qualitative literature focuses on single service evaluations (Paling et al. 2022) or the perceived challenges physiotherapists face within a single service in dealing with people with suspected CES (Paling and Hebron 2021). This survey is the first to examine UK MSK physiotherapist decision making in suspected CES.

Across the 3 clinical vignettes, the data suggested that the strongest predicting factor for a physiotherapist to refer to A&E was the presence of altered bladder function. This is consistent with Wood et al., (2024) who found bladder changes as the most frequently recorded subjective symptom in their retrospective review of 530 cases of back pain. Wood et al., (2024) also calculated sexual dysfunction as having the strongest positive predictive value for CES (25%). Of the 3 case presentations in this study, only vignette 2 presented with reported sexual dysfunction. Those referring to A&E listed this as the second strongest feature influencing their decision.

A systematic review of confirmed *n* = 569 CES cases across 7 studies concluded that spinal red flags appear more specific than sensitive (Dionne et al. 2019). Consequently, prompt diagnostic workup is recommended when a clinician presents with a patient with suspected CES. This is aligned with recommendations made in the most recent GIRFT guidelines (GIRFT. Spinal Surgery 2023). The lower frequency of respondents selecting ‘urgent MRI’ may reflect regional variation in access to imaging and differences in physiotherapy scope of practice. As the survey did not differentiate between advanced practice and non‐advanced practice roles, the influence of MRI‐requesting authority on clinical decision‐making cannot be determined. A proportion of respondents in this survey opted to examine the patient as the next step in their care. It is not possible to delineate in this survey whether the findings of their physical examination would prevent a subsequent A&E referral. One case vignette was presented as a telephone appointment, which suggests that these respondents felt physical examination findings could influence the decision to refer to A&E. Depending on how quickly this assessment could be arranged, it may constitute a delay in care given the time sensitivity of CES (Korse et al. 2017; Metcalfe et al. 2023). GIRFT (2023) states that direct referral is acceptable if an immediate physical examination cannot be arranged. Physical examination findings are useful if available prior to referring to A&E (GIRFT. Spinal Surgery 2023), as it can serve as a means of recognising deterioration in nerve function. Future research should look to clarify the optimal timing of physical assessments in the community following initial virtual appointments to avoid unnecessary delay in A&E referrals, which could be detrimental to patient outcomes.

### 4.1 Handover Methods

More than a third of respondents' preferred handover method was to provide the patient with a written letter (electronic or physical) and refer to A&E. Around a quarter of respondents opted to call the A&E department and supplement this with a handover letter. To the best of the authors' knowledge, there is no standardised handover method outlined within UK guidance for referring to A&E from primary/community care. Current guidelines recommend that patient presentation and assessment findings are ‘documented’ when making an emergency referral (GIRFT. Spinal Surgery 2023). This is to be supported by a physical summary/proforma to be given to the patient if the referral is accepted by secondary care. However, it is not clear if this is in reference to those attending secondary care from A&E or primary care. Future guidelines would benefit from clarity here to reduce unwarranted variation in practice. This may be difficult to implement because of variation in regional pathways resulting from local infrastructure, such as access to emergency imaging or emergency spinal surgery services.

## 5 Rationale for Not Referring to A&E

In cases where clinicians who have opted not to refer to A&E, the key themes include: ‘Already been to A&E’, ‘Assess in Physiotherapy’, ‘Consult a Specialist Doctor’, ‘Urgent/priority MRI’, ‘Outside of Timescales’ and ‘Don't think it is CES’. UK guidelines state that MRI scanning is the current diagnostic gold standard in those with suspected CES, with rectal examination and bladder scanning being insufficient alternatives (GIRFT. Spinal Surgery 2023). The authors recognise the data collection method limits regarding exploring respondents' decision‐making in depth; however, each vignette contains information designed to prompt clinician suspicion.

Regarding Vignette 1, there was clear concern expressed in the comments, leading some to make the decision to investigate via an ‘urgent MRI’. As this is a time sensitive condition, it is important to delineate the definition of ‘urgent’ and ‘emergency’ when used to describe care. GIRFT now defines ‘urgent’ as within 2 weeks, and ‘emergency’ as ‘same day’ (GIRFT. Spinal Surgery 2023), which may help establish a more appropriate use of terms. In the UK, the same day MRI scanning and imaging reports are uncommon outside specialist or emergency settings. To prevent delay, emergency imaging should be accessed through emergency services rather than community services.

For Vignette 2, a patient with high severity (9 weeks) and non‐progressive symptoms (5 weeks) not referring to A&E would be in keeping with the current UK guidance (GIRFT. Spinal Surgery 2023), and the comments reflect the non‐progressive symptoms as a feature in making this decision. An important point to consider is the reported ‘improvement’ in bowel symptoms in someone with a history of irritable bowel syndrome. ‘Change in bowel function’ is a reported feature of concern in these guidelines. Both continence and loss of bowel motility may indicate the loss of CE nerve function (MACSIP 2012), and further information regarding the reasons for the change should be explored.

The comments reflect the complexity of determining the causes of additional symptoms in the presence of other potentially explanatory features. It was recognised that this patient would likely benefit from diagnostic imaging but not through an A&E pathway. This highlighted the importance of ‘safety netting’, a practice outlined in the GIRFT guidance, though the scope of application remains unclear. Should this be considered for all patients with low back pain, only those with radicular pain/radiculopathy, or those who are suspected to have additional non‐MSK features while awaiting for investigation? Given the lack of clear predictive factors for CES development and the potential health and medicolegal implications, future guidelines should clarify when to provide safety net advice in clinical settings.

Vignette 3 outlines a case where respondents did not suspect CES but were still concerned regarding the nature of the underlying cause. Some felt that the age of the patient (65 years old) reduced the likelihood of being CES. Although in line with reported mean ages of onset from lumbar disc herniation, a proportion of cases are described as being associated with lumbar spine stenosis, more common in those more than 65 years old (Comer et al. 2020). The importance of the different structures causing CES is not currently explored in UK guidance, and the authors do not feel this would effectively alter the decision to refer to A&E in cases where CES is suspected.

It is also important to highlight that in some responses, the comments reflect a concern regarding the nature of the patient's pain and therefore recommended that imaging would be arranged by the clinician (for potential malignancy or discitis). As previously highlighted regarding timescales for urgency and for emergency care, caution is required when opting to investigate in the community for cases requiring emergency care. Discitis and malignancy (Metastatic Cord Compression) are also time‐sensitive conditions considered requiring emergency opinions (NHS England 2020).

## 6 Limitations

This study may have been subject to social desirability bias with respondents reporting what they perceive to be the correct clinical action. Furthermore, clinical case scenarios limit respondents' ability to gather additional information which may influence their decision making.

The sampling method likely favoured active social media and interactive discussion board users, introducing sampling bias. The use of the iCSP would help to mitigate this somewhat, as posts lead to email notifications for those who have activated this function.

Low response rates affect the external validity and generalisability of our findings. Despite this, trends across the respondents likely reflect broader patterns among physiotherapists and should not be ignored given the impact of the condition.

## 7 Strengths

This study is the first of its kind to present the clinical reasoning of UK physiotherapists faced with potential serious spinal pathology. The findings of this survey will interest educators at both undergraduate and post graduate levels, clinical leadership teams and patient advocacy groups. Non‐Physiotherapy organisations, such as primary care commissioners and leaders of Secondary/emergency care services, may also find the findings relevant for ensuring timely management of those with suspected CES.

## 8 Future Research

This study provides a framework for future research, which could include (but not be limited to) further surveying of UK physiotherapists since the release of the updated GIRFT guidelines. It would also be useful to consider this or a similar project across other health care professionals, which could highlight areas of unwarranted variation or training needs. Qualitative findings from interviews and/or focus groups could provide deeper insights into clinical reasoning.

## 9 Conclusion

In each vignette within this survey, the largest responses demonstrated a concern regarding the clinical presentation, resulting in most respondents opting for referral to A&E. For those who did not feel direct referral to A&E was appropriate, in each case, several themes emerged which have in part been addressed in the most recent GIRFT guidelines.

This study demonstrates some areas of the variability in practice regarding management options when faced with potentially urgent spinal scenarios. This is a concern given the life changing nature of delayed diagnosis and treatment of CES. Scope remains for future research to clarify the utility of clinical examination in the community ahead of referring to A&E. Given the severity of consequences for those with delayed care for CES, it highlights the need for a more unified approach across UK services.

## 10 Implications for Physiotherapy Practice

Physiotherapists working in UK musculoskeletal services should maintain a high index of suspicion when patients report back pain and associated neurological changes. Where cauda equina syndrome is suspected, clear and timely escalation through established emergency pathways is essential to minimise potential delays in diagnosis and care.

Services may benefit from reviewing local access to emergency imaging and clarifying handover processes to ensure alignment with current national guidance. Structured interprofessional development and consistent safety‐netting practices may further support appropriate and defensible clinical decision‐making.

## Funding

The authors have nothing to report.

## Ethics Statement

Ethical approval was granted by the University of Sunderland Ethics Committee (application number 013539, 27/10/2022).

## Consent

The authors have nothing to report.

## Conflicts of Interest

One author has received speaker fees related to education on cauda equina syndrome and has co‐authored a book on clinical reasoning in suspected cauda equina syndrome. All other authors declare no conflicts of interest.

## Supporting information


Supporting Information S1

## Data Availability

Data are available from the corresponding author upon reasonable request.
