# Supplementary material for: Physiotherapist's Management of Suspected Cauda Equina Syndrome in the United Kingdom: A National Survey
Source: Physiother Res Int. 2026 Mar 17;31(2):e70182. doi: 10.1002/pri.70182 (PMC12993798; doi:10.1002/pri.70182)
Supplement: Supplementary file 1 — Supporting Information S1 [file PRI-31-e70182-s001.docx]

Appendix 1: Completed Standards for Reporting Qualitative Research form

|  | **Standards for Reporting Qualitative Research (SRQR)*** |  |
| --- | --- | --- |
|  | <http://www.equator-network.org/reporting-guidelines/srqr/> |  |
|  |  | **Page/line no(s).** |
| **Title and abstract** | |  |
|  | **Title** –  Physiotherapist's management of suspected Cauda Equina Syndrome in the United Kingdom: A National Survey. | Page 1  Lines 1-4 |
|  | **Abstract** - Summary of key elements of the study using the abstract format of the intended publication; typically includes background, purpose, methods, results, and conclusions | Page 2  Line 11 |
|  |  |  |
| **Introduction** | |  |
|  | **Problem formulation** - Description and significance of the problem/phenomenon studied; review of relevant theory and empirical work; problem statement | Page 3  Line 41 |
|  | **Purpose or research questio**n - Purpose of the study and specific objectives or questions | Page 4  Line 86 |
|  |  |  |
| **Methods** | |  |
|  | **Qualitative approach and research paradigm** - Qualitative approach (e.g., ethnography, grounded theory, case study, phenomenology, narrative research) and guiding theory if appropriate; identifying the research paradigm (e.g., postpositivist, constructivist/ interpretivist) is also recommended; rationale** | Page 5  Line 93 |
|  | **Researcher characteristics and reflexivity** - Researchers’ characteristics that may influence the research, including personal attributes, qualifications/experience, relationship with participants, assumptions, and/or presuppositions; potential or actual interaction between researchers’ characteristics and the research questions, approach, methods, results, and/or transferability | Page 5  Line 110 |
|  | **Context** - Setting/site and salient contextual factors; rationale** |  |
|  | **Sampling strategy** - How and why research participants, documents, or events were selected; criteria for deciding when no further sampling was necessary (e.g., sampling saturation); rationale** | Page 5  Line 101 |
|  | **Ethical issues pertaining to human subjects** - Documentation of approval by an appropriate ethics review board and participant consent, or explanation for lack thereof; other confidentiality and data security issues | Page 5  Line 106 |
|  | **Data collection methods** - Types of data collected; details of data collection procedures including (as appropriate) start and stop dates of data collection and analysis, iterative process, triangulation of sources/methods, and modification of procedures in response to evolving study findings; rationale** | Page 6  Line 136 |
|  | **Data collection instruments and technologies** - Description of instruments (e.g., interview guides, questionnaires) and devices (e.g., audio recorders) used for data collection; if/how the instrument(s) changed over the course of the study | Page 6  Line 139 |
|  | **Units of study** - Number and relevant characteristics of participants, documents, or events included in the study; level of participation (could be reported in results) | Page 7  Line 176 |
|  | **Data processing** - Methods for processing data prior to and during analysis, including transcription, data entry, data management and security, verification of data integrity, data coding, and anonymization/de-identification of excerpts | Page 6  Line 136 |
|  | **Data analysis** - Process by which inferences, themes, etc., were identified and developed, including the researchers involved in data analysis; usually references a specific paradigm or approach; rationale** | Page 6  Line 142 |
|  | **Techniques to enhance trustworthiness** - Techniques to enhance trustworthiness and credibility of data analysis (e.g., member checking, audit trail, triangulation); rationale** | Page 6  Line 142 |
|  |  |  |
| **Results/findings** | |  |
|  | **Synthesis and interpretation** - Main findings (e.g., interpretations, inferences, and themes); might include development of a theory or model, or integration with prior research or theory | Page 7  Line 157 |
|  | **Links to empirical data** - Evidence (e.g., quotes, field notes, text excerpts, photographs) to substantiate analytic findings | Page 7  Line 157 |
|  |  |  |
| **Discussion** | |  |
|  | **Integration with prior work, implications, transferability, and contribution(s) to the field -** Short summary of main findings; explanation of how findings and conclusions connect to, support, elaborate on, or challenge conclusions of earlier scholarship; discussion of scope of application/generalizability; identification of unique contribution(s) to scholarship in a discipline or field | Page 27  Line 297 |
|  | **Limitations** - Trustworthiness and limitations of findings | Page 30  Line 397 |
|  |  |  |
| **Other** | |  |
|  | **Conflicts of interest** - Potential sources of influence or perceived influence on study conduct and conclusions; how these were managed |  |
|  | **Funding** - Sources of funding and other support; role of funders in data collection, interpretation, and reporting | Page 5  Line 108 |
|  |  |  |
|  | *The authors created the SRQR by searching the literature to identify guidelines, reporting standards, and critical appraisal criteria for qualitative research; reviewing the reference lists of retrieved sources; and contacting experts to gain feedback. The SRQR aims to improve the transparency of all aspects of qualitative research by providing clear standards for reporting qualitative research. |  |
|  |  |  |
|  | **The rationale should briefly discuss the justification for choosing that theory, approach, method, or technique rather than other options available, the assumptions and limitations implicit in those choices, and how those choices influence study conclusions and transferability. As appropriate, the rationale for several items might be discussed together. |  |
|  |  |  |
|  | **Reference:** |  |
|  | O'Brien BC, Harris IB, Beckman TJ, Reed DA, Cook DA. **Standards for reporting qualitative research: a synthesis of recommendations.** *Academic Medicine*, Vol. 89, No. 9 / Sept 2014  DOI: 10.1097/ACM.0000000000000388 |  |
|  |  |  |

Appendix 2: Participant information leaflet

**Title**: A survey of UK Physiotherapists regarding the management of patients with suspected Cauda Equina Syndrome.

**Chief Investigator:** 
**Researchers**:
Hello, my name is [removed for manuscript submission], and I am a Senior Lecturer in the Faculty of Health Sciences and Wellbeing, at The University [removed at request of journal]. I am undertaking a research project, and I would like to invite you to take part. Before you decide if you want to please read the following information and discuss it with others if you wish. Please contact me if you have any questions – [removed for manuscript submission]

**What is the purpose of the study?**To establish current practice of UK Physiotherapists when faced with a presentation suspicious of Cauda Equina Syndrome. The findings of this study will be particularly useful in establishing the concordance of practice to current clinical guidance and highlighting areas of focus for education and pathway development.

**Why am I being invited to take part?**You have been invited because I believe you are a chartered Physiotherapist in the UK and consult with patients who complain of lower back pain. To be able to take part though you must be currently practicing Physiotherapy in the UK. Unfortunately, you can't take part if you are not currently practicing as a musculoskeletal Physiotherapist in the UK.

**Do I have to take part?**No, it’s up to you if you want to, or not.

**What would I be asked to do if I chose to take part?**You will be asked to read 3 short clinical case studies and then answer some questions relating to your suspected diagnosis, assessment plan and management plan. This should take around 20 minutes to complete.

**What are the possible disadvantages, or risks, of taking part?**There are no foreseeable disadvantages to taking part.

**What are the possible benefits to taking part?**There are no foreseeable benefits for you taking part, however completion of this study may act as a means of self-reflection and therefore clinical development. The results of this survey could inform the development of clinical guidance and competency development among chartered Physiotherapists.

**What would happen to the information collected about me?**Personal data including special category data obtained for the purposes of this research project is processed lawfully in the necessary performance of scientific or historical research or for statistical purposes carried out in the public interest. Processing of personal data including special category data is proportionate to the aims pursued, respects the essence of data protection and provides suitable and specific measures to safeguard the rights and interests of the data subject in full compliance with the General Data Protection Regulation and the Data Protection Act 2018. The non-identifiable research data will be stored indefinitely on a secure password protected server at University of [removed at request of journal]. This is in case other scientists wish to raise questions about the results that need checking against the dataset. In the event that the study is published in a scientific journal, the non-person identifiable research dataset may be made publicly available (for example, as a supplement to the journal article, or stored on an on-line scientific data repository).

**What would happen if I started, but, changed my mind?**If you decide to stop completing the survey part way through you can close the survey link and your responses will not be used. You have the right to withdraw from participation up until the submission of your questionnaire.

**What happens if there are any problems?**If you are unhappy, or there is a problem, please talk to me. If you remain unhappy, or there is an issue which you do not wish to talk to me about please contact:

[removed at request of journal]

Academic Director of Post Graduate Research
Chair of the Ethics Committee at University of [removed at request of journal] [removed at request of journal]

**Who has approved this study?**This study has been approved by University of [removed at request of journal] Research Ethics Committee.

Thank you for reading this information sheet and for considering whether or not to take part in this study. By completing this survey, you are offering your informed consent to participate.

Appendix 3: Questionnaire

Demographic questions:

1. Are you an HCPC registered Physiotherapist, currently working in the United Kingdon, with a caseload of people seeking your care for Spinal Disorders? Yes/No.
2. Which geographical region would best describe your location:
   1. Scotland
   2. Wales
   3. Northern Ireland
   4. Northeast England
   5. Northwest England
   6. Yorkshire & The Humber
   7. East Midlands
   8. West Midlands
   9. East of England
   10. London
   11. Southeast England
   12. Southwest England
3. How many years qualified are you?
   1. 5 years and under
   2. 5-10 years
   3. 11-15 years
   4. 16-20 years
   5. 21 years +
4. What would best describe your practice setting?
   1. NHS
   2. Independent provider of NHS services
   3. Private practice
   4. Sports Physiotherapy
   5. Occupational Health
5. Do you have a special interest in Spinal disorders? Yes/No.

Following each Vignette, the same questions were asked. Not all questions were completed due to skip logic function, meaning only questions which were applicable to the participant were asked:

1. How suspicious of cauda equina syndrome are you? 0 = no suspicion – 10 = highest suspicion.
2. Which elements of the presentation alter your concern, and how do they alter it? - 5 (most reassured) - +5 (most concerned).
   1. Level of pain
   2. Impact on sleep
   3. Altered bladder function
   4. Altered bowel function
   5. Saddle sensation changes
   6. Bilateral leg symptoms
   7. Sexual dysfunction
   8. Progressive symptoms
3. Does this patient warrant a physical examination? Yes/No.
4. If you were going to physically examine this patient, what would your assessment consist of?
   1. I would assess spinal range of motion
   2. I would assess neurodynamic tests
   3. I would assess lumbar nerve root reflexes
   4. I would assess lumbar nerve root dermatomes
   5. I would assess lumbar nerve root myotomes
   6. I would assess perineal / perianal sensation
   7. I would assess bulbocavernosus reflex
   8. I would assess anal tone
   9. I would assess upper motor neurone lesion tests
   10. I would assess spinal pain on palpation
   11. I would assess spinal segmental movement tests
   12. I would complete abdominal / visceral palpation
5. What would your management plan be today?
   1. Ask the patient to attend A&E today
   2. Ask the patient to attend their nearest walk-in centre
   3. Ask the patient to attend their GP
   4. Direct referral to spinal services
   5. Refer for an Xray
   6. Request an urgent MRI
   7. Request an emergency MRI
   8. Request a routine MRI
   9. See the patient yourself
6. If referring to A&E, how is this communicated with the patient and the service to which you are referring?
   1. I am not referring to A&E
   2. Send patient and ask them to verbally communicate the reason for attendance
   3. Provide MACP (or alternative) CES card to demonstrate your reason for referral
   4. Provide completed service developed written pro forma
   5. Provide written letter as handover (or electronic)
   6. Complete shared electronic record with Spinal Services
   7. Telephone handover to triage clinician
   8. Telephone handover plus written letter
   9. Other – free text on next question
7. If not referring to A&E, please tell us more about your clinical reasoning.
8. What handover method do you use

# Supplementary Table: Physical examination options selected per vignette

|  | **Vignette 1** | **Vignette 2** | **Vignette 3** |
| --- | --- | --- | --- |
| **Answer** | **% (count of answers selected)** | **% (count of answers selected)** | **% (count of answers selected)** |
| **Spinal range of motion** | 12.21% (197) | 12.80% (192) | 12.20% (122) |
| **Neurodynamic tests** | 11.28% (182) | 12.33% (185) | 10.70% (107) |
| **Lumbar nerve root reflexes** | 15.31% (247) | 15.20% (228) | 15.40% (154) |
| **Lumbar nerve root dermatomes** | 15.07% (243) | 14.87% (223) | 15.20% (152) |
| **Lumbar nerve root myotomes** | 15.44% (249) | 14.80% (222) | 15.40% (154) |
| **Perineal / perianal sensation** | 4.28% (69) | 3.40% (51) | 4.20% (42) |
| **Bulbocavernosus reflex** | 0.68% (11) | 0.33% (5) | 0.50% (5) |
| **Anal tone** | 1.61% (26) | 1.07% (16) | 0.80% (8) |
| **Upper motor neurone lesion tests** | 8.49% (137) | 8.00% (120) | 9.90% (99) |
| **Spinal pain on palpation** | 7.87% (127) | 8.60% (129) | 9.40% (94) |
| **Spinal segmental movement** | 2.79% (45) | 3.27% (49) | 2.60% (26) |
| **Repeated movement tests** | 2.79% (45) | 3.87% (58) | 2.30% (23) |
| **Abdominal / visceral palpation** | 2.17% (35) | 1.47% (22) | 1.40% (14) |
| Total | 100% (1613) | 100% (1500) | 100% (1000) |
